# PEGylation improves the therapeutic potential of dimerized translationally controlled tumor protein blocking peptide in ovalbumin-induced mouse model of airway inflammation

**DOI:** 10.1080/10717544.2022.2100511

**Published:** 2022-07-19

**Authors:** Hyeran Seo, Hae-Duck Bae, Haejun Pyun, Bo-Gyu Kim, Sang-Il Lee, Jin-Sook Song, Kyunglim Lee

**Affiliations:** a Graduate School of Pharmaceutical Sciences, College of Pharmacy, Ewha Womans University, Seoul, Korea; b Department of Internal Medicine and Institute of Health Science, Gyeongsang National University School of Medicine and Hospital, Jinju, Republic of Korea; c Data Convergence Drug Research Center, Therapeutics & Biotechnology Division, Korea Research Institute of Chemical Technology, Daejeon, Korea

**Keywords:** PEGylation, dimerized TCTP-binding peptide 2 (dTBP2), dimerized translationally controlled tumor protein (dTCTP), histamine-releasing factor (HRF), allergic airway inflammation

## Abstract

Dimerized translationally controlled tumor protein (dTCTP) initiates a variety of allergic responses in mouse models and that dTCTP-binding peptide 2 (dTBP2) attenuates the allergic inflammation by targeting dTCTP. However, the usefulness of peptide-based drugs is often limited due to their short half-lives, rapid degradation, and high levels of clearance after systemic administration. In this study, we chemically conjugated dTBP2 with 10 kDa polyethylene glycol (PEG) to improve its therapeutic potential. N-terminal mono-PEGylated dTBP2 (PEG-dTBP2) was characterized by *in vitro* bioactivity assay, pharmacokinetics study, and *in vivo* efficacy. When compared to the unmodified dTBP2, PEG-dTBP2 reduced proinflammatory cytokine IL-8 secretion in human bronchial cells by 10 to 15% and increased plasma half-life by approximately 2.5-fold in mice. This study specifically demonstrated that PEG-dTBP2 shows higher inhibitory action against ovalbumin (OVA)-induced airway inflammation in mice compared to dTBP2. Importantly, PEG-dTBP2, when administered once at 1 mg/kg, significantly reduced the migration of inflammatory cells and the levels of cytokines in the bronchoalveolar lavage fluids as well as OVA-specific IgE levels in serum. In addition, the degree of goblet cell hyperplasia and mucus secretion were significantly attenuated in the PEG-dTBP2 group compared with the control group. These results suggest that PEG-dTBP2 can be considered a potential candidate drug for regulating allergic inflammation.

## 1. Introduction

Translationally controlled tumor protein (TCTP), also known as histamine releasing factor (HRF), p21, p23, and fortilin, is a highly conserved protein found in all eukaryotic organisms (Bommer & Thiele, 2004). TCTP has been associated with the regulation of several biological processes, including cell cycle progression and anti-apoptotic activity as well as Na, K-ATPase suppression (Gachet et al., 1999; Li et al., 2001; Jung et al., 2004). TCTP also acting as an extracellular protein, is able to modulate the release of various cytokines in several different cell types involved in immune functions (Schroeder et al., 1997). These cell types include bronchial epithelial cells (IL-8, GM-CSF release), basophils (IL-4, IL-13 release), B cells (IL-, IL-8 release), and T-cells (inhibition of IL-2, IL-13 release) (Kashiwakura et al., 2012; Macdonald, 2012). Extracellular TCTP is elevated in nasal lavages, bronchoalveolar lavage fluids (BALFs), and skin blister fluids of allergic patients (Warner et al., 1986; MacDonald et al., 1987; Lichtenstein, 1988; Macdonald, 2012), suggesting its clinical significance in human allergic diseases. Inhibition of extracellular TCTP, particularly in the dimeric form (dTCTP), reduced the release of histamine and Th2 cytokines (IL-4, IL-13) from mast cells and basophils and ameliorated symptoms in a mouse model of food-allergic enteropathy (Ando et al., 2017). Also, inhibition of dTCTP reduced immune cell infiltration and Th2 cytokines in the lung as well as mucus production in a mouse model of ovalbumin (OVA)-induced allergy (Pyun et al., 2018), both of which suggest that blockage or inhibition of dTCTP should potentially be a useful approach for treating allergic diseases.

In previous study, we have demonstrated that dTCTP which exists in the sera of atopic and atopic/asthmatic patients and BALFs from mice with airway inflammation, but not monomeric TCTP, is the moiety responsible for its cytokine-like activities and its role in the regulation of allergic reactions (Kim et al., 2009). Therefore, we speculated that blockade of the dTCTP might have potential use in the therapy of allergic diseases. dTCTP-binding peptide-2 (dTBP2) binds to dimeric, but not the monomeric form of TCTP, was shown to inhibit TCTP’s cytokine-like activity *in vitro*. In addition, the administration of dTBP2 ameliorated allergic symptoms, such as asthma, atopy, and rhinitis in mice (Kim et al., 2011; Jin et al., 2017; Cho et al., 2021).

However, dTBP2, like other synthetic peptides, is rapidly degraded *in vivo* compared to big molecular biopharmaceuticals, such as proteins and antibodies. Therefore, it is necessary to improve its *in vivo* stability. The conjugation of polyethylene glycol (PEG) to therapeutic peptides has been shown to be one of the most successful strategies for improving their pharmacokinetics and pharmacodynamics by decreasing blood clearance, protecting from enzymatic degradation, and reducing renal clearance (Diao & Meibohm, 2013). Therefore, we prepared N-terminal mono-PEGylated dTBP2 (PEG-dTBP2) and explored its therapeutic usefulness. We found that the PEG-dTBP2 did exhibit improved therapeutic efficacy without losing its bioactivity in the mouse disease models.

## 2. Materials and methods

### 2.1. Materials and reagents

Recombinant dTCTP was cloned into the pRSET-A expression vector with a His-tag and was expressed in the *Escherichia coli* strain BL21 (DE3) pLysS (Merck, Germany). The resulting protein was purified using Ni-NTA agarose (QIAGEN, Germany) and ion exchange chromatography with HiTrap Q column (GE Healthcare Bio-Sciences Corp). dTBP2 (WYVYPSM) was synthesized by Peptron, Inc. (Daejeon, South Korea). The purity of the peptide was >90%. Methoxy PEG-propionaldehyde (mPEG-aldehyde; MW 10 kDa) and sodium cyanoborohydride (NaCNBH_3_) were purchased from Sigma (St. Louis, MO). OVA and aluminum hydroxide were purchased from Thermo Fischer Scientific (Waltham, MA). IL-4, IL-5, IL-8, and OVA-specific IgE ELISA assay kit was purchased from Biolegend (San Diego, CA). IL-13 ELISA assay kit was purchased from R&D Systems (Minneapolis, MN). All the chemicals used in this study were of analytical grade.

### 2.2. Preparation of N-terminal Mono-PEG-dTBP2

**Figure 1.**
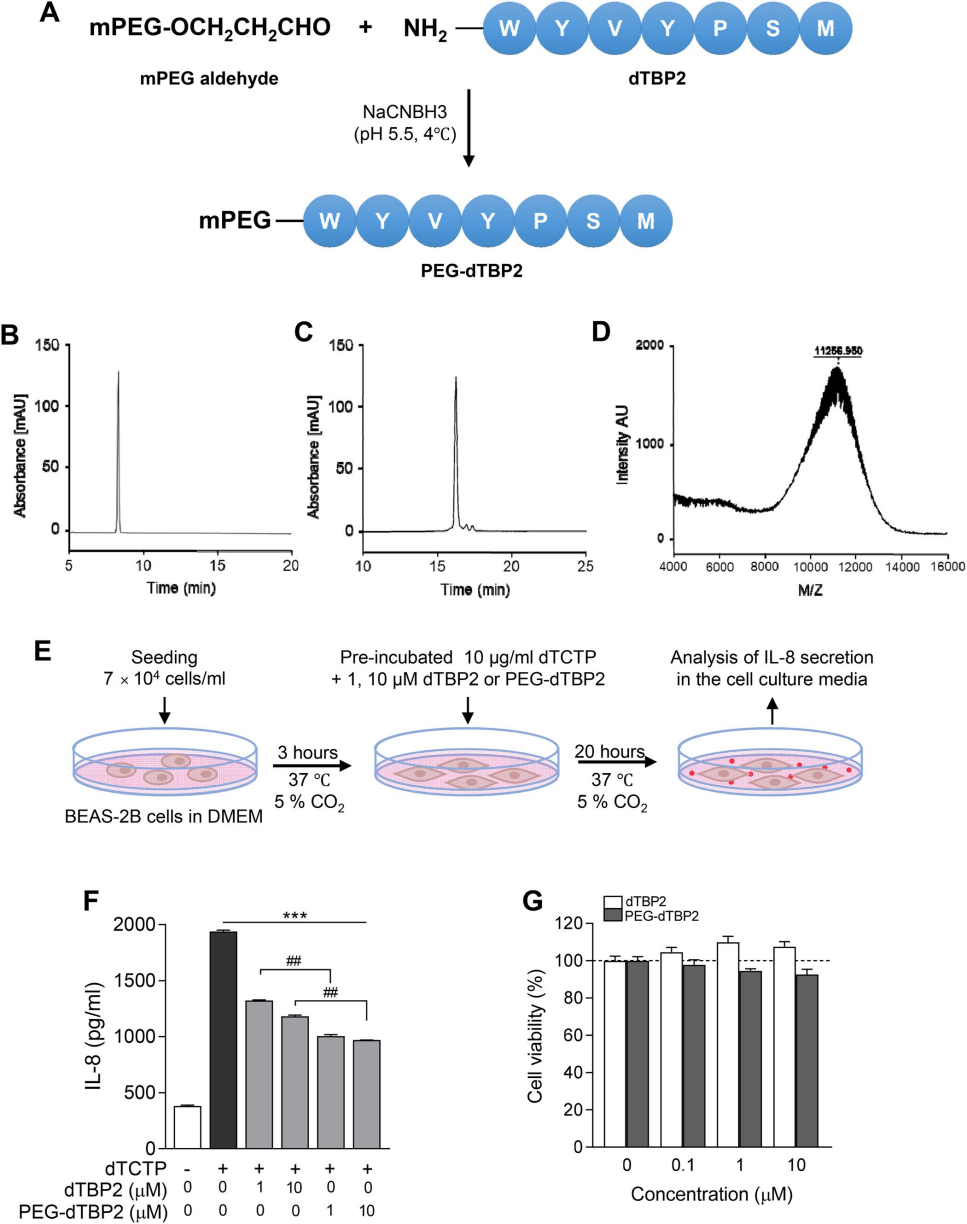
Characterization of PEG-dTBP2. (A) A scheme showing the synthesis of PEG-dTBP2. (B) Reversed-phase HPLC chromatogram of dTBP2. (C) Reversed-phase HPLC chromatogram of reaction mixture between PEG and dTBP2. (D) MALDI-TOF mass spectrum of purified PEG-dTBP2. (E) Scheme of *in vitro* experiment on BEAS-2B cells. (F) The ability of dTBP2 and PEG-dTBP2 to inhibit IL-8 secreted by dTCTP (10 μg/ml) in BEAS-2B cells. Each bar represents mean ± SEM (*n* = 3). ***p < 0.0001 compared with dTCTP alone. ^##^*p* < 0.001 versus dTBP2. (G) Relative cytotoxicities of dTBP2 and PEG-dTBP2 in BEAS-2B cells. Vertical bars represent means ± SEM (*n* = 3).

PEG-dTBP2 was prepared by BiopolyMed Inc. (Seoul, Korea) as illustrated in Figure 1A. The PEGylation was conducted as previously described with minor modifications (Na et al., 2003). N-terminal mono-PEGylation of dTBP2 was performed with mPEG-aldehyde (MW 10 kDa) in the presence of 20 mM NaCNBH_3_ in 0.1 M sodium acetate buffer at pH 5.5. The reaction was performed with a molar ratio of 1:10 (dTBP2:mPEG-aldehyde) at 4 °C overnight with gentle mixing. The PEG-dTBP2 was isolated from the reaction mixture using an Amicon ultra-15 ml centrifugal filter (MW cutoff 10 kDa, Millipore). The PEG-dTBP2 was further purified by reversed-phase HPLC on a Gemini C18 column (4.6 mm i.d. × 250 mm, 5 µm particle size; Phenomenex, Torrance, CA). Gradient elution was performed at a flow rate of 1 ml/min with buffer A (0.1% TFA in DW) and buffer B (0.1% TFA in acetonitrile), using a 25-50% B linear gradient over 30 min. Chromatograms were recorded at the wavelength of 220 nm. The molecular weight of PEG-dTBP2 was determined using MALDI-ToF/MS.

### 2.3. Cell culture and analysis of IL-8 in cell supernatant

BEAS-2B, human bronchial epithelial cells, were purchased from the American Type Culture Collection (ATCC, CRL-9609) and cultured in Dulbecco’s Modified Eagle’s Medium (DMEM, WelGENE) at 37 °C, 5% CO2, and a humidified atmosphere.

Cell culture to measure IL-8 was carried out as previously described (Kim et al., 2009). The indicated amounts (1 and 10 μM) of dTBP2 and PEG-dTBP2 were pre-incubated with recombinant dTCTP (10 μg/ml) for 15 min, then added to the cells in 1% penicillin-streptomycin/DMEM, and the cultures incubated for 20 h. IL-8 secreted into the medium was measured by ELISA using a commercial kit.

### 2.4. Cell viability assay

BEAS-2B cells (1 × 10^4^ cells/well) were incubated in 96-well plates with various concentrations of dTBP2 and PEG-dTBP2 for 24 h. Cell viability was measured using the 3-(4,5-dimethylthiazol-2-yl)-2,5-diphenyl tetrazolium bromide (MTT) assay (Sigma) according to the manufacturer’s instructions. The percentage of viable cells was calculated using the equation: cell viability (%) = (mean absorbance in test wells/mean absorbance in control wells) × 100.

### 2.5. Animals

Male ICR mice and female BALB/c mice were purchased from Orient Bio, Inc. (Gyeonggi, Korea). They were maintained with free access to water and standard rodent feed. All animal studies were approved by Ewha Womans University’s Institutional Animal Care and Use Committee (IACUC 20-002). The photoperiod was set at a 12 L:12D lighting schedule. The room temperature was maintained at 23 ± 2 °C.

### 2.6. Metabolic stability and pharmacokinetics of PEG-dTBP2

dTBP2 and PEG-dTBP2 were examined for *in vitro* metabolic stability using liver microsomes obtained from humans and mice. This examination was performed as previously described with minor modifications (Cho et al., 2017). The test peptides (final concentration: 1 μM) with human or mouse liver microsomes (final concentration: 0.5 mg/ml) in 100 mM potassium phosphate buffer (pH 7.4) were pre-incubated at 37 °C for 5 min. The reactions were initiated by adding an NADPH regenerating system (BD Biosciences). After 30 min of incubation, the reactions were terminated by the addition of 10 times the volume of ice-cold acetonitrile with buspirone hydrochloride as an internal standard. Samples were then centrifuged at 8,000 × g for 15 min at 4 °C, and the supernatants were analyzed by liquid chromatography-mass spectrometry (LC-MS/MS).

Pharmacokinetic studies of dTBP2 and PEG-dTBP2 were conducted in ICR male mice with 3 animals in each group at a dose of 10 mg/kg. dTBP2 and PEG-dTBP2 were administered by intravenous route. Blood samples were collected from the orbital sinus at 0.083, 0.25, 0.5, 1, 2, 4, and 8 h after dosing with dTBP2 or PEG-dTBP2 in mice. Plasma samples were obtained after centrifugation at 4000 × g for 25 min. Ten times the volume of ice-cold acetonitrile was added to plasma and mixed well for 5 min. The samples were centrifuged at 8,000 × g for 15 min at 4 °C, and the supernatants were analyzed by LC-MS/MS.

The pharmacokinetic parameters were calculated from the concentration-time data using the non-compartmental analysis tool of Phoenix WinNonlin software (version 6.4, Pharsight, St. Louis, MO).

### 2.7. Mouse model of OVA-induced allergic airway inflammation

Sixty female BALB/c mice, 6–7 weeks of age, were randomly divided into five groups (12 mice per group) and intraperitoneally sensitized on days 1 and 14 with 50 μg OVA emulsified in 2 mg aluminum hydroxide in a total volume of 200 μl. The control group of naïve mice received the same volume of PBS for the duration of the experiment. Two weeks after the second sensitization, the animals were challenged with 200 μg of OVA through intranasal administration under light anesthesia on days 28, 30, 32, and 34. Each group was treated with PBS, 1 mg/kg of dexamethasone (Dex), dTBP2, or PEG-dTBP2 by intraperitoneal injection on day 32. On day 35, the mice were sacrificed and BALF, lung tissue, and serum were collected for further analysis.

### 2.8. BALF collection and inflammatory cell count

Mice were anesthetized, and the trachea was excised, and cannulated with a polyethylene tube. The lungs were lavaged with 0.8 ml of PBS four times, and 0.6 ml of BALF was obtained. The collected lavage fluid was cooled on ice and centrifuged at 1,000 rpm for 10 min at 4 °C. The supernatant was used for ELISA assays. The pellets were resuspended in 0.1 ml PBS, and the total inflammatory cell numbers were quantified using a Hemavet (Drew Scientific Inc., Oxford, CT).

### 2.9. Preparation of lung tissue homogenates

After lavage, the right lung lobe was isolated and stored at −80 °C until required. The lung tissue was homogenized in 50 mM Tris-HCl buffer (pH 7.4) containing 150 mM NaCl, 1 mM EDTA, 0.25% deoxycholate, and a protease inhibitor (Sigma) using a tissuelyser II (Qiagen). The lung homogenates were centrifuged at 10,000 × g for 10 min, and the supernatants were collected and used for ELISA assay.

### 2.10. Histological analysis

The left lung lobe was fixed in 4% formaldehyde, embedded in paraffin, and sectioned at a thickness of 4 μm. The sections were stained with hematoxylin and eosin (H&E) to detect infiltration of inflammatory cells and with periodic acid-Schiff (PAS) staining for the analysis of goblet cells hyperplasia/mucus production. The H&E stained sections were scored for the presence of inflammatory cells using the following scale: (0) = absent, (1) = rare, (2) = mild, (3) = moderate, (4) = severe. Scoring was performed by 6 observers in a blinded manner. Also, the area of H&E stained cells was quantified in the selected field, and the area of PAS-positive cells per bronchiole was expressed as the % of the total epithelial area using ImageJ software (National Institute of Health, Bethesda, MD).

### 2.11. Statistical analysis

Statistical analysis was performed using GRAPHPAD^TM^ PRISM version 8.0 for Windows (GraphPad Software, San Diego, CA). Statistical significance was determined using Student’s two-tailed unpaired *t-*test for comparisons between two groups. For more than 3 groups, a one-way analysis of variance (ANOVA) was followed by Tukey’s test. Error bars were expressed as the mean ± the standard error of the mean (SEM) of 10 to 12 mice per group. Significant differences between means were defined as *p* < 0.05.

## 3. Results and discussion

### 3.1. Synthesis and in vitro bioactivity of PEG-dTBP2

Site-specific N-terminal PEGylation of dTBP2 was performed using mPEG-aldehyde (10 kDa) as a PEGylating agent in the presence of NaCNBH_3_ at pH 5.5 (Figure 1A). Reversed-phase HPLC analysis confirmed the fraction of PEG-dTBP2 in the reaction product to be approximately 90.5% (Figure 1C). The PEG-dTBP2 fraction was identified by measuring the molecular weights by MALDI-TOF MS. The measured mass of PEG-dTBP2 revealed the molecular weight of PEG-dTBP2 to be approximately 11.257 (Figure 1D). This PEGylation resulted in the production of mono-PEG-dTBP2 at a molar ratio of 1:1 (dTPB2:mPEG-aldehyde).

The increased molecular mass of therapeutic peptides by PEGylation led to improved water solubility and metabolic stability as well as reduced renal clearance, leading to the enhanced residence time of therapeutic peptides in the body. However, it is recognized that PEGylation of a therapeutic peptide may negatively affect their binding affinity to target proteins due to steric hindrance of the interaction (Imura et al., 2007).dTCTP induces the secretion of the proinflammatory cytokine IL-8 through transcriptional and post-transcriptional regulation of the NF-κB pathway in BEAS-2B cells (Lee & Lee, 2018). We also previously showed that dTBP2 increases IκB expression reduced by dTCTP, and dTBP2 selectively inhibited the nuclear localization of NF-κB p65, resulting in dTBP2 suppressing dTCTP-mediated IL-8 secretion (Cho et al., 2021). Therefore, we first examined and confirmed whether the PEG chain inter-molecularly hampers the binding to dTCTP and thus does ose not block the cytokine-like activity of dTCTP. To evaluate the inhibitory effects of PEG-dTBP2 on the dTCTP-induced secretion of IL-8 in BEAS-2B cells, these cells were incubated with dTCTP plus various amounts (0, 1, and 10 µM) of PEG-dTBP2 for 20 h in 1% penicillin–streptomycin/DMEM. Native dTBP2, reported in a previous study (Kim et al., 2011), was used as a control.

As shown in Figure 1F, the secretion of IL-8 increased after 20 h incubation with dTCTP. In contrast, both native dTBP2 and PEG-dTBP2 attenuated the cytokine-like activity of dTCTP in BEAS-2B cells and significantly reduced the IL-8 secretion in a dose-dependent manner compared to treatment with dTCTP (*p* < 0.0001). For example, 1 and 10 µM of PEG-dTBP2 reduced IL-8 secretion by 45% and 50%, respectively, and the same concentrations of native PEG-dTBP2 reduced the level of IL-8 to 30% and 40%, respectively. When compared to native dTBP2, the PEG-dTBP2 significantly reduced IL-8 secreted from BEAS-2B cells stimulated by dTCTP (Figure 1F, *p* < 0.001).

After PEG conjugation of dTBP2, improvement of water solubility and stability of dTBP2 led to a marked improvement in its bioactivity. We also found that PEG-dTBP2 had no significant effect on BEAS-2B cell viability up to a dose of 10 µM (Figure 1G). These findings suggest that conjugation of dTBP2 with PEG with a molecular weight of 10 kDa did not affect its bioactivity.

### 3.2. Metabolic stability and pharmacokinetics of PEG-dTBP2

**Figure 2.**
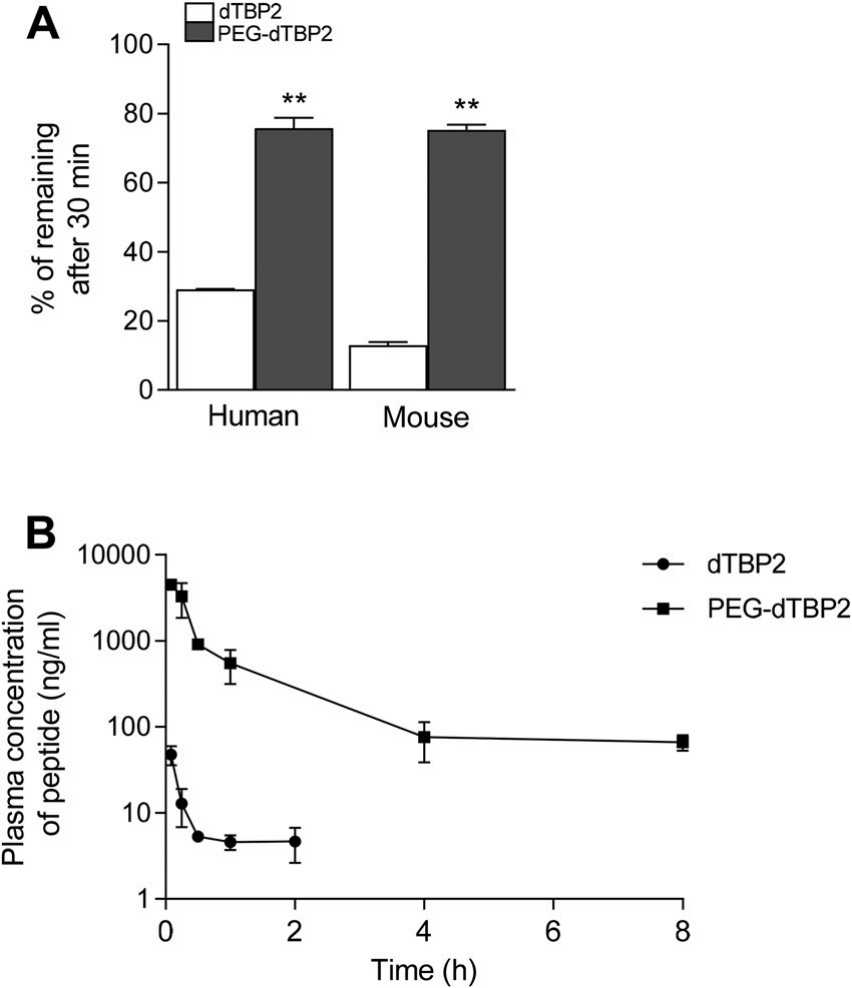
Metabolic stability and pharmacokinetics of PEG-dTBP2. (A) % of dTBP2 and PEG-dTBP2 remaining after 30 minutes of incubation with human or mouse liver microsomes. Vertical bars represent means ± SEM. **p < 0.001 compared with dTBP2 by Student’s two-tailed unpaired *t*-test. (B) Plasma concentration-time profiles of dTBP2 and PEG-dTBP2 in mice. The results are expressed as means ± SEM (*n* = 3 per group).

To clarify the stabilizing effect of PEGylated peptide, native dTBP2 and PEG-dTBP2 were incubated with human or mouse liver microsomes for 30 min at 37 °C and subjected to LC-MS/MS analysis. As shown in Figure 2A, native dTBP2 was degraded with 30% of intact peptides remaining after 30 min incubation with human liver microsomes, whereas 75% of PEG-dTBP2 remained at the same time. The metabolic stability of PEG-dTBP2 was found to be 2.5- and 5-fold better than native dTBP2 in human and mouse liver microsomes, respectively. This enhanced metabolic stability could provide a prolonged duration of pharmacological action. Conjugation with a much higher molecular weight of PEG may contribute to better stabilization.

We carried out a pharmacokinetic study of native dTBP2 and PEG-dTBP2 to evaluate the effects of PEGylation on the pharmacokinetic properties in mice. The plasma concentrations of native dTBP2 and PEG-dTBP2 after intravenous dosing (10 mg/kg) are shown in Figure 2B, and the pharmacokinetic parameters calculated by Phoenix WinNonlin software are shown in Table 1. Terminal T_1/2_ and AUC of PEG-dTBP2 increased by about 2.54-fold and 116-fold more than dTBP2 (*p* < 0.01), respectively, whereas the volume of distribution and clearance values decreased. These results demonstrate that PEGylation greatly improves the pharmacokinetic properties of dTBP2.

**Table 1.** Pharmacokinetics parameters of dTBP2 and PEG-dTBP2 following intravenous administration in male mice.

Parameter	dTBP2 (10 mg/kg)	PEG-dTBP2 (10 mg/kg)
T_1/2_	1.01 ± 0.25	2.57 ± 1.11
AUC_last_ (μg·hr/ml)	0.02 ± 0.004	3.20 ± 0.63**
AUC_0-∞_ (μg·hr/ml)	0.03 ± 0.01	3.49 ± 0.61**
CL (L/kg/hr)	409.01 ± 98.89	3.06 ± 0.57***
V_ss_ (L/kg)	487.21 ± 168.28	6.35 ± 2.57

Data are expressed as mean ± SEM (*n* = 3). Data are statistically significant ^**^p < 0.01, ^***^p < 0.001, compared with dTBP2 group. T_1/2_ terminal half-life, AUC areas under the plasma concentration-time curve, CL total clearance, Vss volume of distribution of steady state.

### 3.3. PEG-dTBP2 suppresses Th2 cytokines expression in airway inflammation

Recently, we reported that daily intraperitoneal injection of dTBP2 at a high dose (5 mg/kg for 7 days) attenuates airway inflammation in a mouse model of allergic asthma by inhibiting dTCTP activity (Cho et al., 2021). To determine whether PEG-dTBP2 may be more potent in reducing allergic airway inflammation than native dTBP2, both dTBP2 and PEG-dTBP2 were individually administered in a single-dose intraperitoneal injection after the third intranasal OVA challenge. PBS-challenged mice were used as controls. Dexamethasone, belonging to the glucocorticoid family, was used as a positive control for anti-inflammatory and immunosuppressive substances.

**Figure 3.**
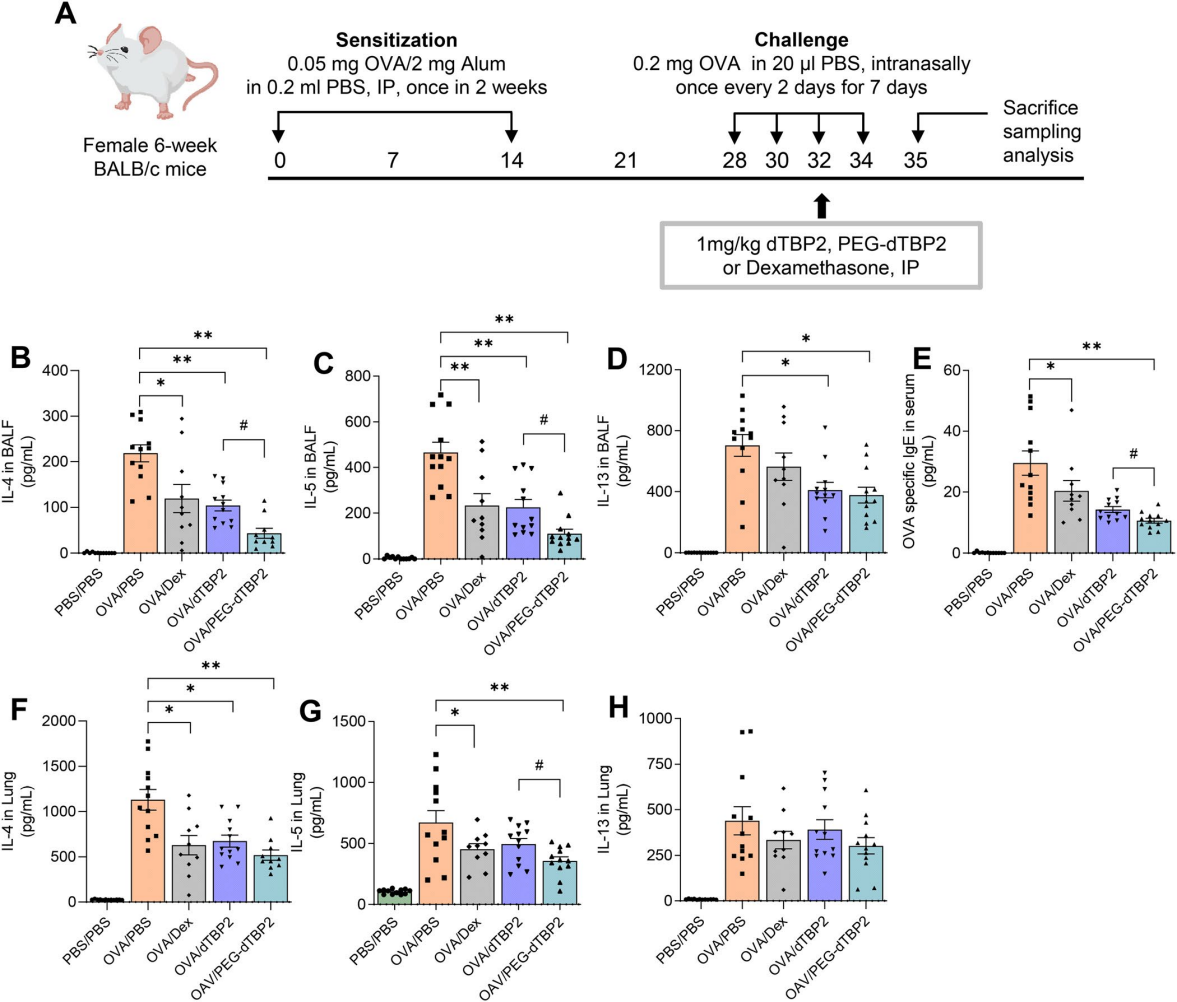
PEGylation enhances the anti-inflammatory efficacy of dTBP2 in asthma. (A) Schedule for OVA-induced asthma mouse model and treatment with PEG-dTBP2. Levels of IL-4, IL-5, and IL-13 in BALF (B-D) and lung homogenates (F-H) were measured, and the level of OVA-specific IgE (E) in serum are measured by ELISA. The results are expressed as means ± SEM (*n* = 10-12 per group). *p < 0.05, **p < 0.001 OVA/PBS group versus other treatment groups by One-way Tukey’s test. ^#^*p* < 0.05 OVA/dTBP2 group versus OVA/PEG-dTBP2 by Student’s two-tailed unpaired *t*-test. IP, intraperitoneal. IN, intranasal. Dex, dexamethasone.

Th2 cells migrate to the airways and locally produce Th2 cytokines in asthma. Levels of Th2 cytokines in BALB/c mice correlate with the clinical severity of asthma. IL-5 promotes eosinophil activation in the lung. IL-13 and IL-4 are associated with the basic properties of asthma, such as migration of eosinophils into tissues, goblet cell maturation, mucus secretion, and bronchial hypersensitivity (Liang et al., 2017). Therefore, we first determined whether PEG-dTBP2 influenced Th2 cytokine expression in BALF. As shown in Figure 3B–D, IL-4, IL-5, and IL-13 levels of BALF, compared with the PBS-challenged mice markedly increased in the OVA-challenged group. Compared with the OVA-challenged group, the treatment groups showed decreased levels of IL-4, IL-5, and IL-13 with a down-regulation of Th2 cells. Compared to native dTPB2, PEG-dTBP2 showed significant decreases in IL-4 (Figure 3B) and IL-5 (Figure 3C) in the BALF (*p* < 0.05), while IL-13 tended to decrease. We also evaluated cytokine levels in the lung homogenates of treated mice (Figure 3F–H). The levels of IL-4, IL-5, and IL-13 remarkably increased by OVA challenge, these increases were suppressed by Dex, dTBP2, or PEG-dTBP2 treatment. Compared to that of dTPB2, the level of IL-5 significantly decreased (Figure 3G, *p* < 0.05) by PEGylation of dTBP2, and the levels of IL-4 (Figure 3F) and IL-13 (Figure 3H) also tended to non-significantly decrease. These data suggest that PEG-dTBP2 exhibits superior attenuation of Th2 allergic responses in the lung compared with dTBP2.

Because IgE mediates mast cell and basophil degranulation by FcεRI cross-linking upon allergen recognition, the serum levels of allergen-specific IgE are crucial for the development of allergic responses (Hamilton et al., 2021). Therefore, we determined the concentration of OVA-specific IgE in the serum. Among the treatment groups, serum OVA-specific IgE levels were significantly reduced in mice treated with PEG-dTBP2 compared to the control group (Figure 3E, *p* < 0.01), which suggests that PEG-dTBP2 reduces the IgE-mediated allergic response.

### 3.4. PEG-dTBP2 reduces the numbers of inflammatory cells in BALF

**Figure 4.**
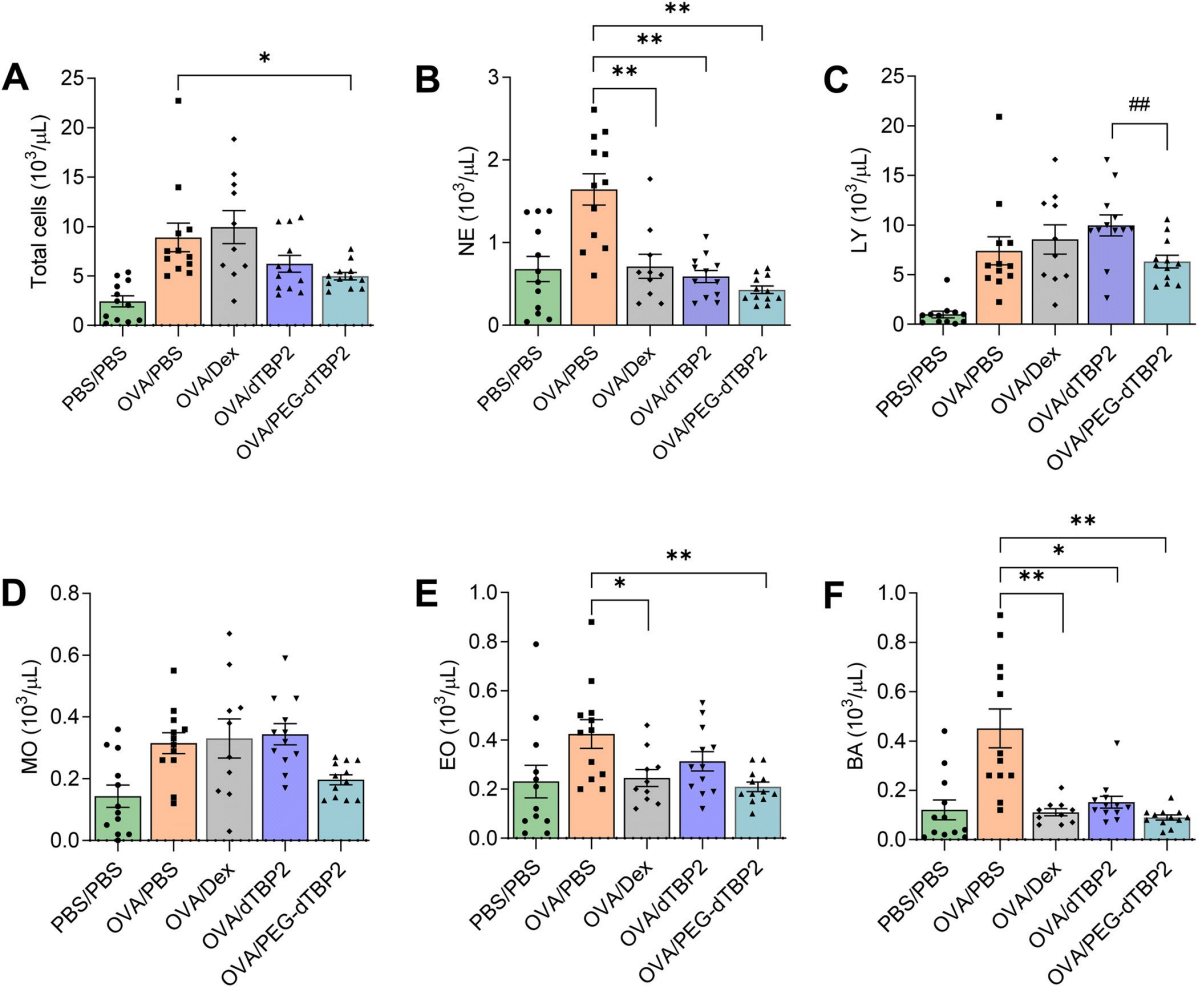
PEG-dTBP2 decreases the recruitment of total inflammatory cells (A), neutrophils (B), lymphocytes (C), monocytes (D), eosinophils (E), and basophils (F) in BALF. The values are expressed as means ± SEM (*n* = 10-12). *p < 0.05, **p < 0.001 OVA/PBS group versus other treatment groups by One-way Tukey’s test. ^##^*p* < 0.001 OVA/dTBP2 group versus OVA/PEG-dTBP2 by Student’s two-tailed unpaired *t*-test. Dex, dexamethasone.

To further determine the anti-inflammatory effects of PEG-dTBP2, BALF cells were collected, and the number of infiltrated cells was analyzed 24 h after the last OVA challenge. The infiltration of inflammatory cells in Th2 cell response to allergen exposure is one of the classical signs of asthma. As shown in Figure 4, OVA-challenged mice resulted in increased infiltration of neutrophils, lymphocytes, monocytes, eosinophils, and basophils, also determine by total cells, compared to PBS-challenged mice. The dTBP2-treated group showed reduced the infiltration of inflammatory cells (6.2 ± 2.8 × 10^3^/µl), and that was further decreased in the PEG-dTBP2-treated group (5.0 ± 1.2 × 10^3^/µl). Thus, treatment with dTBP2 and PEG-dTBP2 resulted in 30% and 45% inhibition in the infiltration of inflammatory cells in the BALF compared with the OVA-challenged group. When compared to the OVA-challenged group, the mice from the PEG-dTBP2-administered group showed a significantly reduced number of total cells and eosinophils while the mice from the dTBP2-administered group were not significantly different (Figure 4A and E). Thus, the PEG-dTBP2 is a more effective inhibitor of lung inflammation and particularly potent in preventing infiltration of eosinophils, which are believed to play important roles in the development of asthma exacerbations.

Treatment with Dex (positive control) did not significantly suppress the numbers of lymphocytes, and monocytes when compared to the OVA group. Choi *et al* used a dose of 10 mg/kg of Dex every 2 days for 11 days to test the effects of Dex treatment on airway inflammation (Choi et al., 2021). They also used Dex as a positive control and showed that the numbers of eosinophils, macrophages, lymphocytes, and neutrophils in BALF decreased significantly. However, the single dose of Dex (1 mg/kg) was used in the present study and this was lower than that used in the previous study. This may be the reason why there is no difference in the total leukocytes, lymphocytes, and monocytes between the OVA/PBS and OVA/Dex groups.

### 3.5. Histopathological changes induced in the animal lungs are ameliorated by PEG-dTBP2 administration

Allergens induce pathophysiological changes in the lungs in asthma. Airway hypersensitivity is caused by hypertrophy and proliferation of smooth muscle cells, and goblet cells in the airway secrete mucus and promote airway remodeling (Aikawa et al., 1992; Rogers, 2007).

**Figure 5.**
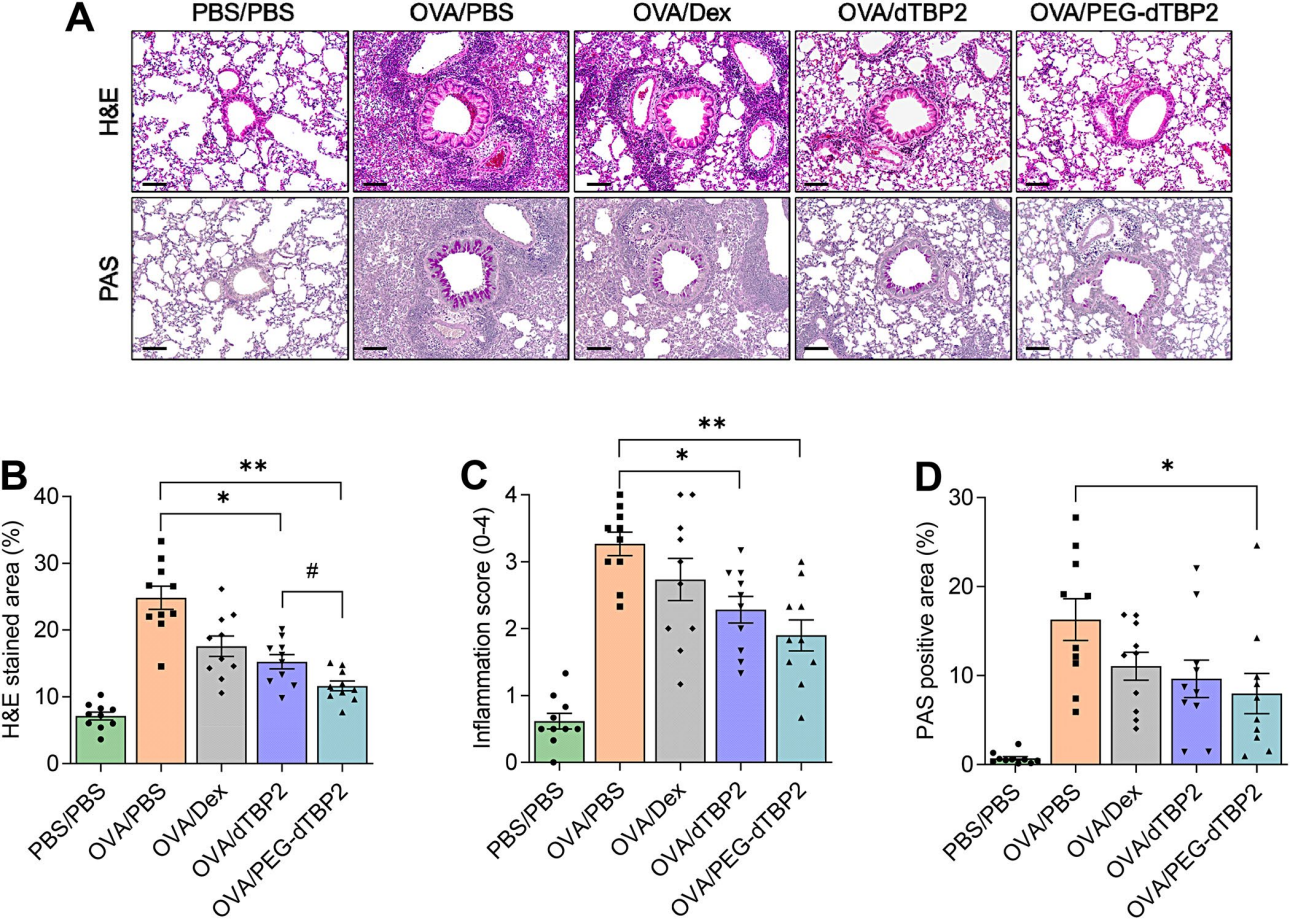
PEG-dTBP2 reduces inflammation and goblet cell hyperplasia in the ovalbumin-induced mouse model of asthma. (A) H&E and PAS staining of lung section (scale bar, 100 μm). (B) Percentage of H&E stained area and (C) inflammation score within a selected field for inflammatory cell infiltration. (D) Percentage of PAS-positive cells per bronchiole in total epithelial area each mouse for goblet cell hyperplasia and mucus production. Vertical bars represent means ± SEM (*n* = 10). *p < 0.05, **p < 0.001 compared with OVA/PBS group by One-way Tukey’s test. ^#^*p* < 0.05 OVA/dTBP2 group versus OVA/PEG-dTBP2 by Student’s two-tailed unpaired *t*-test. OVA, ovalbumin. Dex, dexamethasone.

To characterize the effects of PEG-dTBP2 on the pathological features in the OVA-induced allergic airway inflammation mouse model, lung sections were stained with H&E and PAS. The lung tissue of OVA-stimulated mice showed numerous inflammatory cells accumulated around the bronchovascular bundles and bronchioles, also many PAS-positive mucus-producing cells were observed in the airway epithelium (Figure 5A). When airway inflammation was scored in a blinded manner, there was no significant difference between the dTBP2 and PEG-dTBP2 administrated groups (Figure 5C), although there was a statistical difference when measuring the H&E-stained area indicating inflammatory cell infiltration (Figure 5B, *p* < 0.05). In addition, since PEG-dTBP2 showed a decrease in the proliferation of goblet cells compared to the OVA-challenged group (Figure 5D, *p* < 0.05), PEG-dTBP2 tended to exhibit more effectiveness in attenuating airway inflammation and mucus production compared with the dTBP2.

Patients with severe asthma remain poorly controlled despite using high doses of corticosteroids in conjunction with long-acting beta-agonists (Dreher & Müller, 2018). Furthermore, the use of oral corticosteroids is suspected to cause severe adverse reactions including bone fractures, hyperglycemia, susceptibility to infections, and hypertension (Manson et al., 2009). Therefore, new treatments with new therapeutic agents are needed to reduce or replace the use of corticosteroids for improving lung function of patients with severe uncontrolled asthma.

Toward this end, we isolated by screening a phage-displayed random peptide library, a 7mer peptide, that specifically binds to dTCTP that we called dTBP2 [15]. We found that dTBP2 binds to the helix α2 corresponding to the 89-110 sequence of TCTP, indicating that helix α2 plays an important role in the cytokine-like function of dTCTP (Lee et al., 2020). However, dTBP2 exhibited an extremely short plasma half-life after intravenous administration.

Molecular modification of therapeutic peptides, such as by, covalent conjugation with PEG, or other chemical modifications including cyclization, and D-amino acid substitution represent strategies to improve the *in vivo* half-life and stability of peptide-based drugs. PEG molecules are highly water-soluble, nontoxic, non-immunogenic, and are approved by FDA. PEG-conjugated therapeutic peptides or proteins have been reported to exhibit preclinical and clinical properties superior to those of their corresponding unmodified parent molecules by prolonging half-life and low volume of distribution (Bumbaca et al., 2019).

In this study, we first generated PEG-conjugated dTBP2 and confirmed their enhanced therapeutic potential, superior *in vitro* bioactivity, metabolic stability, and *in vivo* efficacy in our asthma animal models compared to that of unmodified dTBP2. For these reasons, PEG-dTBP2 was explored as a potential drug candidate for allergic inflammatory diseases. We previously proposed that dTCTP may bind to its putative receptor on the target cell and trigger cytokine release (Kim et al., 2013). We speculate that PEG-dTBP2 may bind to dTCTP and interrupt the binding of dTCTP with its putative receptor, effectively inhibiting the dTCTP-induced cytokine release and inflammatory response. The mechanism underlying the anti-inflammatory efficacy of PEG-dTPB2 is not clear and needs to be investigated in future studies.

## 4. Conclusion

dTCTP plays an important role in allergic inflammation by inducing the secretion of histamine and cytokines essential for the late phase of allergic responses, thereby initiating inflammatory reactions responsible for allergic responses. Substances that inhibit these reactions by binding with and incapacitating dTCTP can be the keys to the therapy of allergic diseases. In the present study, a dTCTP binding peptide, dTBP2 7mer peptide, was conjugated with PEG at the N-terminus of dTBP2 and the resulting PEG-dTBP2 conjugate was shown to exhibit improved metabolic stability and increased half-life relative to the parent peptide. In the mouse allergic airway inflammation models, PEG-dTBP2 shows enhanced anti-inflammatory effects compared to unconjugated dTBP2. These results suggest that PEG-dTBP2 can be considered a potential candidate drug for regulating allergic inflammation.

## References

[CIT0001] Aikawa T, Shimura S, Sasaki H, et al. (1992). Marked goblet cell hyperplasia with mucus accumulation in the airways of patients who died of severe acute asthma attack. Chest 101:916–21. 1555462 10.1378/chest.101.4.916

[CIT0002] Ando T, Kashiwakura J-I, Itoh-Nagato N, et al. (2017). Histamine-releasing factor enhances food allergy. J Clin Invest 127:4541–53. 29130935 10.1172/JCI96525 PMC5707161

[CIT0003] Bommer U-A, Thiele B-J. (2004). The translationally controlled tumour protein (TCTP). Int J Biochem Cell Biol 36:379–85. 14687915 10.1016/s1357-2725(03)00213-9

[CIT0004] Bumbaca B, Li Z, Shah DK. (2019). Pharmacokinetics of protein and peptide conjugates. Drug Metab Pharmacokinet 34:42–54. 30573392 10.1016/j.dmpk.2018.11.001 PMC6378135

[CIT0005] Choi WS, Kang HS, Kim HJ, et al. (2021). Vinpocetine alleviates lung inflammation via macrophage inflammatory protein-1β inhibition in an ovalbumin-induced allergic asthma model. Plos ONE 16:e0251012. 33914833 10.1371/journal.pone.0251012 PMC8084130

[CIT0006] Cho H, Kim HK, Oh A, et al. (2021). dTBP2 attenuates severe airway inflammation by blocking inflammatory cellular network mediated by dTCTP. Biomed Pharmacother 144:112316. 34628164 10.1016/j.biopha.2021.112316

[CIT0007] Cho W, Koo JY, Park Y, et al. (2017). Treatment of sepsis pathogenesis with high mobility group box protein 1-regulating anti-inflammatory agents. J Med Chem 60:170–9. 28001381 10.1021/acs.jmedchem.6b00954

[CIT0008] Diao L, Meibohm B. (2013). Pharmacokinetics and Pharmacokinetic–Pharmacodynamic Correlations of Therapeutic Peptides. Clin Pharmacokinet 52:855–68. 23719681 10.1007/s40262-013-0079-0

[CIT0009] Dreher M, Müller T. (2018). Add-on therapy for symptomatic asthma despite long-acting beta-agonists/inhaled corticosteroid. Tuberc Respir Dis (Seoul) 81:1–5. 29256220 10.4046/trd.2017.0102 PMC5771741

[CIT0010] Gachet Y, Tournier S, Lee M, et al. (1999). The growth-related, translationally controlled protein P23 has properties of a tubulin binding protein and associates transiently with microtubules during the cell cycle. J Cell Sci 112:1257–71. 10085260 10.1242/jcs.112.8.1257

[CIT0011] Hamilton JD, Harel S, Swanson BN, et al. (2021). Dupilumab suppresses type 2 inflammatory biomarkers across multiple atopic, allergic diseases. Clin Exp Allergy 51:915–31. 34037993 10.1111/cea.13954 PMC8362102

[CIT0012] Imura Y, Nishida M, Ogawa Y, et al. (2007). Action mechanism of tachyplesin I and effects of PEGylation. Biochim Biophys Acta 1768:1160–9. 17320042 10.1016/j.bbamem.2007.01.005

[CIT0013] Jin X-H, Lim J, Shin D, et al. (2017). Dimerized translationally controlled tumor protein-binding peptide ameliorates atopic dermatitis in NC/Nga mice. Int J Mol Sci 18:256. 10.3390/ijms18020256 PMC5343792 28134765

[CIT0014] Jung J, Kim M, Kim M-J, et al. (2004). Translationally controlled tumor protein interacts with the third cytoplasmic domain of Na,K-ATPase α subunit and inhibits the pump activity in HeLa cells. J Biol Chem 279:49868–75. 15383549 10.1074/jbc.M400895200

[CIT0015] Kashiwakura J-I, Ando T, Matsumoto K, et al. (2012). Histamine-releasing factor has a proinflammatory role in mouse models of asthma and allergy. J Clin Invest 122:218–28. 22133880 10.1172/JCI59072 PMC3248297

[CIT0016] Kim M, Chung J, Lee C, et al. (2011). A peptide binding to dimerized translationally controlled tumor protein modulates allergic reactions. J Mol Med (Berl) 89:603–10. 21384150 10.1007/s00109-011-0740-8

[CIT0017] Kim M, Maeng J, Lee K. (2013). Dimerization of TCTP and its clinical implications for allergy. Biochimie 95:659–66. 23104268 10.1016/j.biochi.2012.10.007

[CIT0018] Kim M, Min HJ, Won HY, et al. (2009). Dimerization of translationally controlled tumor protein is essential for its cytokine-like activity. PLoS ONE 4:e6464. 19649253 10.1371/journal.pone.0006464 PMC2715101

[CIT0019] Lee H, Kim M-S, Lee J-S, et al. (2020). Flexible loop and helix 2 domains of TCTP are the functional domains of dimerized TCTP. Sci Rep 10:197. 31932619 10.1038/s41598-019-57064-9 PMC6957494

[CIT0020] Lee H, Lee K. (2018). Dimerized translationally controlled tumor protein increases interleukin-8 expression through MAPK and NF-κB pathways in a human bronchial epithelial cell line. Cell Biosci 8:13. 10.1186/s13578-018-0214-6 PMC5819651 29484169

[CIT0021] Li F, Zhang D, Fujise K. (2001). Characterization of fortilin, a novel antiapoptotic protein. J Biol Chem 276:47542–9. 11598139 10.1074/jbc.M108954200

[CIT0022] Liang P, Peng S, Zhang M, et al. (2017). Huai Qi Huang corrects the balance of Th1/Th2 and Treg/Th17 in an ovalbumin-induced asthma mouse model. Bioscience Rep 37(6). 10.1042/BSR20171071 PMC5741832 29162668

[CIT0023] Lichtenstein LM. (1988). Histamine-releasing factors and IgE heterogeneity. J Allergy Clin Immunol 81:814–30. 2453542 10.1016/0091-6749(88)90936-0

[CIT0024] Macdonald SM. (2012). Potential role of histamine releasing factor (HRF) as a therapeutic target for treating asthma and allergy. J Asthma Allergy 5:51–9. 23055753 10.2147/JAA.S28868 PMC3461606

[CIT0025] MacDonald SM, Lichtenstein LM, Proud D, et al. (1987). Studies of IgE-dependent histamine releasing factors: heterogeneity of IgE. J Immunol 139:506–12. 2439589

[CIT0026] Manson SC, Brown RE, Cerulli A, Vidaurre CF. (2009). The cumulative burden of oral corticosteroid side effects and the economic implications of steroid use. Respir Med 103:975–94. 19372037 10.1016/j.rmed.2009.01.003

[CIT0027] Na DH, Murty SB, Lee KC, et al. (2003). Preparation and stability of poly(ethylene glycol) (PEG)ylated octreotide for application to microsphere delivery. AAPS PharmSciTech 4:E72. 15198567 10.1208/pt040472 PMC2750665

[CIT0028] Pyun H, Kang U, Seo EK, Lee K. (2018). Dehydrocostus lactone, a sesquiterpene from Saussurea lappa Clarke, suppresses allergic airway inflammation by binding to dimerized translationally controlled tumor protein. Phytomedicine 43:46–54. 29747753 10.1016/j.phymed.2018.03.045

[CIT0029] Rogers DF. (2007). Physiology of airway mucus secretion and pathophysiology of hypersecretion. Respir Care 52:1134–1149. 17716382

[CIT0030] Schroeder JT, Lichtenstein LM, MacDonald SM. (1997). Recombinant histamine-releasing factor enhances IgE-dependent IL-4 and IL-13 secretion by human basophils. J Immunol 159:447–52. 9200485

[CIT0031] Warner JA, Pienkowski MM, Plaut M, et al. (1986). Identification of histamine releasing factor(s) in the late phase of cutaneous IgE-mediated reactions. J Immunol 136:2583–7. 2419443

